# Large Semi-Membrane Covered Composting System Improves the Spatial Homogeneity and Efficiency of Fermentation

**DOI:** 10.3390/ijerph192315503

**Published:** 2022-11-23

**Authors:** Xiaoxi Sun, Guangqun Huang, Yuanping Huang, Chen Fang, Xueqin He, Yongjun Zheng

**Affiliations:** Laboratory of Biomass and Bioprocessing Engineering, College of Engineering, China Agricultural University, Beijing 100083, China

**Keywords:** agricultural engineering, cow manure, trough aerobic composting, turning, environment

## Abstract

Homogenous spatial distribution of fermentation characteristics, local anaerobic conditions, and large amounts of greenhouse gas (GHGs) emissions are common problems in large-scale aerobic composting systems. The aim of this study was to examine the effects of a semi-membrane covering on the spatial homogeneity and efficiency of fermentation in aerobic composting systems. In the covered group, the pile was covered with a semi-membrane, while in the non-covered group (control group), the pile was uncovered. The covered group entered the high-temperature period earlier and the spatial gradient difference in the group was smaller compared with the non-covered group. The moisture content loss ratio (5.91%) in the covered group was slower than that in the non-covered group (10.78%), and the covered group had a more homogeneous spatial distribution of water. The degradation rate of organic matter in the non-covered group (11.39%) was faster than that in the covered group (10.21%). The final germination index in the covered group (85.82%) was higher than that of the non-covered group (82.79%) and the spatial gradient difference in the covered group was smaller. Compared with the non-covered group, the oxygen consumption rate in the covered group was higher. The GHG emissions (by 30.36%) and power consumption in the covered group were reduced more significantly. The spatial microbial diversity of the non-covered group was greater compared with the covered group. This work shows that aerobic compost covered with a semi-membrane can improve the space homogeneity and efficiency of fermentation.

## 1. Introduction

According to data from the “National Dairy Industry and Technology System (2020)”, China currently has more than four thousand large-scale dairy farms, approximately 50% of which have more than five hundred dairy cows. Rapid development of farming provides not only great amounts of milk and beef, but also large amounts of cattle manure. In recent years, the annual production of cattle manure in China has reached 1.38 billion tons [1]. Unreasonable disposal of cattle manure may cause water, air, and soil pollution. It is important to use this dairy manure properly, promote the recycling of agricultural waste resources, and improve the agricultural environment. 

Aerobic composting has been widely used in the recovery, stabilization, and volume reduction of solid waste in dairy manure, because of its high capacity for treatment and low operational cost [2]. Major large-scale aerobic composting in China is carried out in stacked troughs and by using regular mechanical turning or forced aeration, which are commonly used to provide O_2_ during aerobic composting [3,4,5]. Wang et al. [5] showed that forced aeration reduced the spatial differences in oxygen content. He et al. [4] also reported that the duration of coupled regulative turning and forced aeration (O_2_ > 5%) accounted for 80% of the total duration of the large-scale trough aerobic composting. However, there were still spatial gradient differences in O_2_, CO_2_, CH_4_, and N_2_O concentrations in the compost piles [6]. The difference in gas concentration gradient in the pile is mainly attributed to the large size of the compost pile. A large composting pile may cause a long and complicated transportation path of oxygen from the bottom to the top. Accordingly, a spatial gradient difference in the composting pile may lead to local anaerobic, uneven fermentation and greenhouse gas (GHGs) emissions [7], which need to be improved.

In recent years, semi-permeable membrane-covered aerobic composting, an improved forced ventilation static composting technology with energy-saving and emission-reduction features, has captured researchers’ attention [8]. Composting piles are wrapped by a semi-permeable membrane with the circulation of forced air from the bottom. The membrane “swells” due to the difference in pressure inside and outside the membrane. There is a micro-positive pressure environment in the composting pile under the membrane, which may help oxygen penetrate the composting particles, improve the local anaerobic condition, reduce GHGs emissions, and improve the efficiency of composting [9,10,11]. Ma et al. [9] demonstrated that functional membranes decreased the possibility of global warming by 16.97% and improved the relative abundance of *Actinobacteria*. Fang et al. [12] suggested that carbon dioxide (CO_2_) and methane (CH_4_) were reduced by 78.68% and 99.97%, respectively, in the membrane-covered group compared with the control group. Fang et al. [13] found that membrane-covered composting improved the oxygen utilization rate and inhibited the anaerobic bacterial genus *Hydrogenispora* and archaea order *Methanobacteriales*. The abovementioned studies compared the effect of membrane-covered versus non-covered composting on the aerobic composting process. In large-scale composting, however, to date, there are very few studies showing whether a semi-membrane covering will affect the spatial gradient difference of composting.

In addition, aerobic composting is a biochemical reaction process dominated by microbial activities, which is affected by composting substrates and environmental parameters (i.e., the composition, temperature, and gas composition of the compost) [14,15,16,17]. The heat and mass transfer rate during the aerobic fermentation process may differ in large composting piles [18,19]. The physical spatial gradient of composting piles may directly affect the gas transport, water infiltration, and microbial activity [20,21,22]. Additionally, it may lead to spatial differences in temperature, gas concentration, organic matter degradation rate, and compost maturity during the composting process [5,7]. Management methods such as flipping and additives may also affect the spatial difference to some extent [2,23,24]. However, the mechanisms of covered semi-permeable membrane on the spatial gradient difference of physical and chemical indicators, gas emissions, and microbial distribution are still unclear.

Therefore, whether membrane-covered composting will affect the spatial gradient difference of physicochemical properties, temperature, and GHGs emissions needs to be investigated. In addition, the differences in the microbial community between the upper and bottom layers of compost piles in membrane-covered composting also needs to be explored. The aim of the present study is to examine the effect of a membrane-covered aerobic composting system on the abovementioned parameters of interest. 

## 2. Methods and Materials

### 2.1. Raw Materials and Mixing Ratio

**Figure 1:**
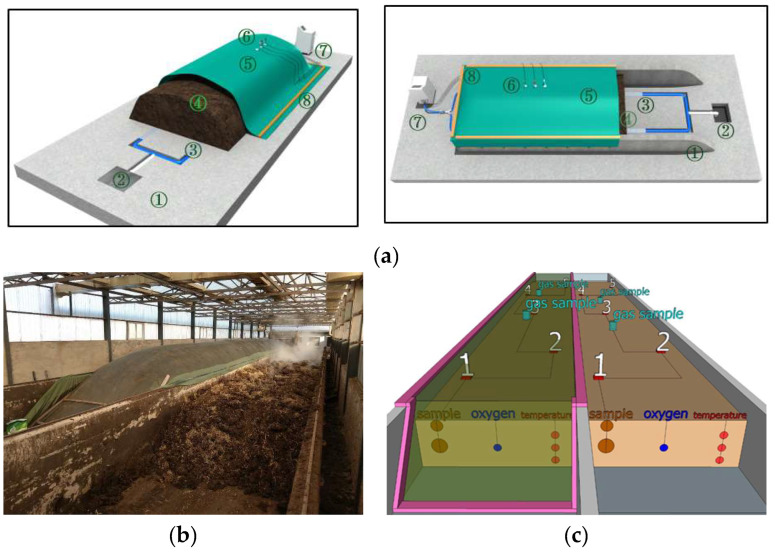
Schematic diagram of the structure of semi-membrane covering aerobic composting system (**a**), the real composting conditions (**b**), and the sample points (**c**). Notes for (**a**): 1. Hardened Ground; 2. Drainage Ditch; 3. Aeration Pipeline; 4. Compost Piles; 5. Functional Membrane; 6. Testing Sensors (Temperature, Oxygen, Pressure); 7. Control System; 8. Membrane Sealing.

Dairy manure (DM) was obtained from a dairy farm in the area near the composting plant. The DM’s moisture content (MC) was 67.47% ± 1.44% and organic matter (OM) was 53.66% ± 1.44%. Cornstalk (CK) was provided by Agricultural Machinery Cooperative in Miyun District, Beijing. The CK was collected from the field by the baler, and the particle size was about 10 cm. The CK’s MC was 29.94% ± 7.09% and OM was 80.76% ± 3.06%. The compounding ratio of DM and CK was DM:CK = 10:1 (based on weight) and then 40% compost (powdery) was added to get a proper mixed raw composting material. The compost’s MC was 18.57% ± 1.88% and OM was 31.57% ± 1.50%. The membrane-covered aerobic composting (the covered group) and the non-covered composting (the non-covered group) were performed separately in two composting tanks. Around 300–350 tons of raw composting material was loaded into the composting tank. Both troughs had a tank (height: 44 m; width: 3.85 m; length: 1.8 m) and a turning machine (Figure 1a). A functional membrane was used (Qingdao Zhiteng Technology Co., Ltd., Qingdao, China) and its main parameters are shown in Table 1. A membrane sealing device for trough turning was constructed using turner tracks (Figure 1b,c). The experiment was conducted at Beilangzhong Organic Fertilizer Factory (Shunyi District, Beijing) by using two parallel compost troughs.

### 2.2. Experiment Design

In Figure 1a, the membrane-covered aerobic composting system mainly consisted of a compost tank, functional membrane, centrifugal fan, air distribution pipeline, flow meter, control device, and sensors. Forced aeration and turning were combined for supplying oxygen. Two centrifugal fans were used (maximum air volume: 51.6 m^3^/min) and the aeration mode was adjusted by self-developed intelligent control equipment. During the composting process, the air volume of the two tanks was set as 39.92 m^3^/min (36 HZ) on Days 1–17 and 33.59 m^3^/min (30 HZ) on Days 18–36. The aeration mode was interval (1 min on and 5 min off). Compost piles were turned on Days 1, 17, and 27. 

### 2.3. Real-Time Collection Data of Temperature and Oxygen Content and Sample Collection

In Figure 1c, the temperature was monitored by PT100 temperature sensors at three different layers (upper: 0 cm, middle: 35 cm, bottom: 70 cm) of composting piles. The oxygen concentration was detected by Biogas 5000 (Gaetch, London, UK) to monitor the middle layer of composting piles. Compost data were recorded by self-developed intelligent monitoring and control equipment [25]. The temperature and oxygen concentration data were collected every day. The gas samples were collected every two days using the static box method with 500 mL air bags placed at two “circles” between sampling points 3 and 5 [4]. Ammonia was measured by a colorimetric tube (GASTEC 3La/3M, Kanagawa, Japan). GHGs (CH_4_, N_2_O, and CO_2_) were measured by gas chromatography (Agilent 7890, Santa Clara, CA, USA) [18]. Composting solid samples were collected every four days by a soil sampler at the upper and bottom layers of five points (Figure 1c) from the front to the back of the pile. Additionally, each collected solid sample was approximately 1 kg and stored in a refrigerator at −20 °C for testing. Another 20 g solid samples used for microbial analysis were stored at −80 °C. 

### 2.4. Physicochemical Methods of Samples

The moisture content (MC) was determined by drying the samples in a stove at 105 °C until constant weight. The organic matter (OM) was determined by using the standard method TMECC 05.07A. The carbon/nitrogen ratio was determined by an elemental analyzer (Elementar Vario ELIII, Augsburg, Germany) [12]. The determination of pH and electrical conductivity (EC) was based on TMECC 04.11A. The germination index (GI) was measured as Selim et al. [26] described. The abovementioned assays were repeated twice.

### 2.5. DNA Extraction and Illumina MiSeq Sequencing

The Omega Soil DNA Kit (Norcross, GA, USA) was used to extract microbial DNA from compost samples. Then, a NanoDrop 2000 UV–vis spectrophotometer (Thermo Scientific, Waltham, MA, USA) was used to determine DNA concentration and purity. The bacterial 16S rRNA gene was amplified with primers 338F (5′-AMACMAACGGGAGGCAGCAG-3′) and 806R (5′-GGAMAACHVGGGTWTMAAAT-3′) by a thermocycler PCR system (GeneAmp 9700, ABI, Portsmouth, VA, USA) [27]. The Miseq PE300 platform (Illumina, San Diego, CA, USA) was used to measure bacterial communities.

### 2.6. Statistical Analysis

Data processing and graphing were performed using Excel 2016 (Microsoft Windows, Washington, DC, USA) and Origin 2017 (OriginLab, Northampton, MA, USA). Considering the accuracy of the static box method to measure the gas emission rate, Origin 2017 (OriginLab) was used to calculate the linearity error of the gas emission rate by the linear model [8]. SPSS 20 was used to analyze the differences in the indicators among the groups.

Operational taxonomic units (OTUs) were clustered with 97% similarity cutoff using UPARSE (version 7.1 http://drive5.com/uparse/, accessed on 2 July 2022.). The taxonomy of each 16S rRNA gene sequence was analyzed by the RDP Classifier algorithm (http://rdp.cme.msu.edu/, accessed on 2 July 2022.) against the 16S rRNA database (Silva v132) using a confidence threshold of 70%. Alpha diversity index was used and different random sampling carried out by using mothur and creating graphs using R language tools. Ace was an index used to estimate the number of OTUs in a community, proposed by Chao. Hierarchical clustering analysis was performed according to the beta diversity distance matrix [28] and the UPGMA (Unweighted Pair-group Method with Arithmetic Mean, unweighted group average method) algorithm was used to construct a tree structure to visualize the degree of similarity or difference in community composition in different environmental samples. The beta diversity distance matrix was calculated by the Qiime algorithm of UPGMA software and then a picture tree was created by R language (version 3.3.1). The species composition of different groups (or samples) at each taxonomic level (such as domain, kingdom, phylum, class, order, family, genus, species, OTU, etc.) can be drawn as a bar chart by using the R language (version 3.3.1) tool. Multilevel Species Discriminant Analysis LEfSe with linear discriminant analysis (LDA) was also used for discovering high-dimensional biomarkers and revealing genomic features, including genes, metabolism, and taxonomy, which was used to distinguish two or more biological conditions (or groups) [29].

## 3. Results and Discussion

### 3.1. Spatial Distribution of Temperature in the Compost Pile

During the whole aerobic process, there was no significant difference in temperature between the membrane-covered and non-covered groups (*p* = 0.426). 

**Figure 2:**
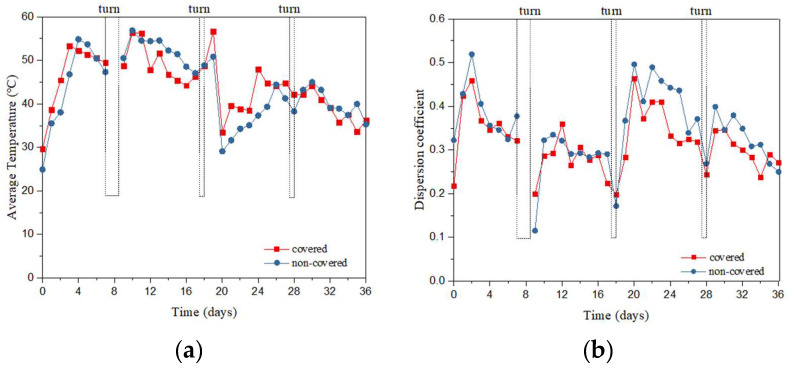

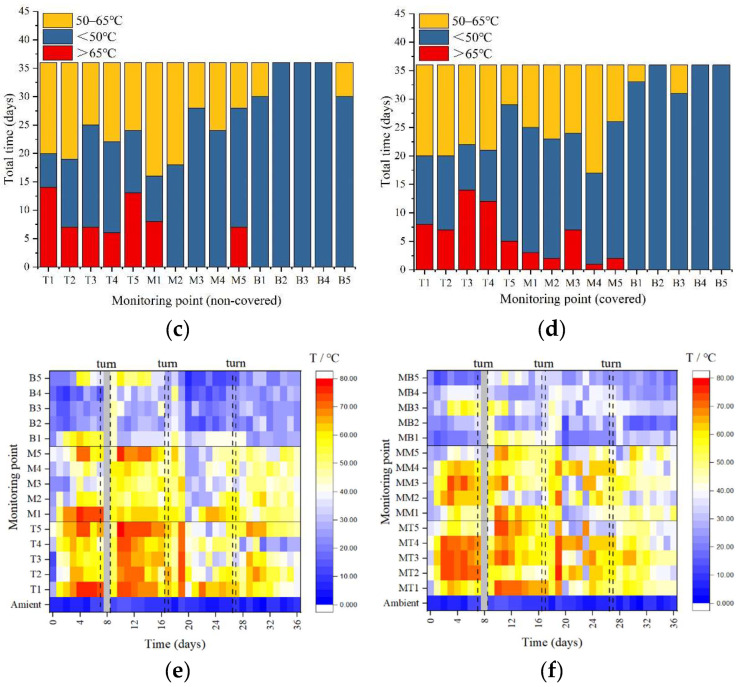
Average temperature (**a**) and dispersion coefficient of temperature (**b**) during aerobic composting; proportion of total time in different temperature ranges at different monitoring points in the non-covered group (**c**) and the covered group (**d**); temperature spatial distribution in the non-covered group (**e**) and the covered group (**f**). B1–5, the five monitoring points at the bottom of the pile in the non-covered group; M1–5, the five monitoring points in the middle of the pile in the non-covered group; T, the five monitoring points at the top of the pile in the non-covered group; MB1–5, the five monitoring points at the bottom of the pile in the covered group; MM1–5, the five monitoring points in the middle of the pile in the covered group; MT, the five monitoring points at the top of the pile in the covered group.

In Figure 2a,b the average temperature in the two groups increased by over 50 °C during week 1. On Days 7, 17, and 27, there was a small increase in temperature during turning. It was shown that turning affected the chemical and biological parameters [30]. In Figure 2b, there was a significant difference in temperature spatial distribution (top vs. middle vs. bottom) within the covered group or within the non-covered group during whole aerobic composting (*p* < 0.001). The spatial distribution difference in temperature within the covered group was smaller than that within the uncovered group. This indicated that the membrane cover reduced the heat loss in the compost piles and reduced the temperature fluctuation caused by other factors such as the environment, which was consistent with a previous study [31].

In Figure 2c,d, there were no significant differences in the proportion of high-temperature time at monitoring points between the covered group and non-covered group (*p* > 0.1). Before the first turning, the monitoring point with a temperature above 65 °C accounted for 20% in the covered group and 15% in the non-covered group. After the first turning, the composting plies with a temperature above 65 °C accounted for 23.70% and 17.04% in the covered group. This indicated that the membrane cover could improve the fermentation efficiency of the composting piles to a certain extent by increasing the oxygen utilization rate [11]. In summary, the membrane covering influenced the composting temperature by the high efficiency of fermentation during the high-temperature period, smaller differences in spatial distribution of temperature, and heat dissipation rate at the bottom. The membrane reduced heat dissipation and latent heat of vaporization and water vapor condensed to preserve heat inside the composting piles [32].

### 3.2. Spatial Distribution of Physicochemical Properties of Compost Samples

#### 3.2.1. Moisture Content (MC)

**Figure 3:**
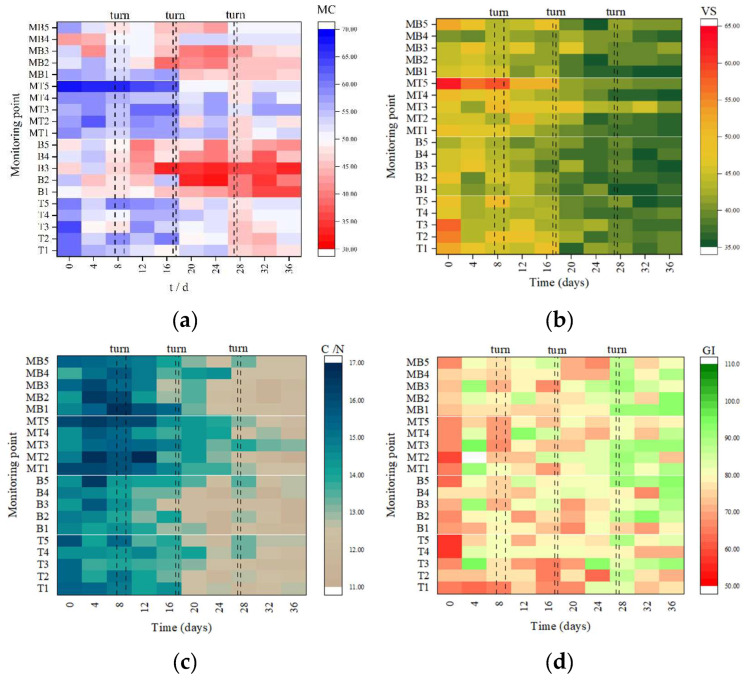

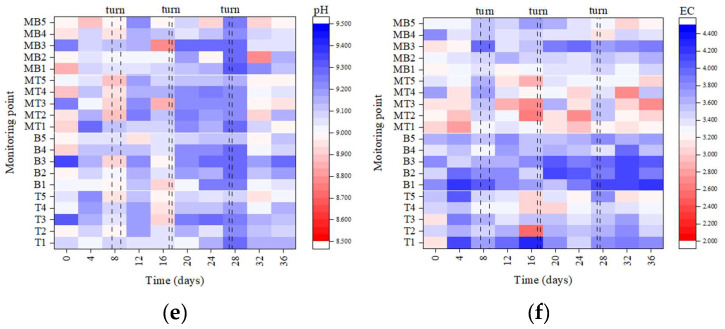
Spatial distribution of moisture content (MC) (**a**), violates solid (VS) (**b**), the ratio of carbon to nitrogen (C/N) (**c**), Germination Index (GI) (**d**), pH (**e**), and electrical conductivity (EC) (**f**) at different monitoring points during aerobic composting. B1–5, the five monitoring points at the bottom of the pile in the non-covered group; T, the five monitoring points at the top of the pile in the non-covered group;). MB1–5, the five monitoring points at the bottom of the pile in the covered group; MT, the five monitoring points at the top of the pile in the covered group.

The moisture content (MC) decreased during the whole composting period in the two groups. The MC decreased from 56.15% ± 6.38% at the beginning to 50.24% ± 3.62% at the end in the covered group and from 55.64% ± 6.11% to 44.86% ± 6.91% in the non-covered group. Membrane covering slowed down the water loss in the composting piles. In Figure 3a, there was a significant difference in MC between the upper and bottom layers in the covered and non-covered groups during aerobic composting (*p* < 0.001). The MC in the upper layer was higher than that in the bottom layer, which was due to water migration by aeration and evaporation [33]. However, the difference in the spatial distribution of MC in the pre-composting covered group was greater than that in the non-covered group. The difference in the spatial distribution of MC in the covered group after Day 8 was lower than that in the non-covered group. A large amount of water could not be volatilized by the membrane covering in time due to the rapid increase in the fermentation rate in the covered group in the early stage of composting and this accumulated in the upper layer of the pile [34]. Then, the composting degradation slowed down and the water vapor under the membrane covering condensed back and permeated downward, resulting in a more homogenous and higher spatial distribution of MC in the covered group than in the non-covered group. In addition, turning alleviated the spatial difference in MC in the composting piles.

#### 3.2.2. Violate Solid (VS) and Ratio of Carbon to Nitrogen (C/N)

The VS in the non-covered group decreased from 49.06% ± 5.08% to 37.67% ± 1.39%. In the covered group, the VS decreased from 48.11% ± 6.12% to 37.90% ± 2.24%. Overall, the degradation rate in the non-covered group was slightly faster than that in the covered group. In Figure 3b, the difference in VS between the upper and bottom layers of the covered and non-covered groups was significant (*p* for non-covered = 0.009, *p* for covered = 0.031). The degradation rate of VS was directly related to microbial activity and temperature, O_2_ concentration, MC, and other factors [35]. The micro- positive pressure environment may promote the penetration of O_2_ into the compost particles, improve the oxygen consumption rate, and further increase the overall degradation rate of the compost pile in the covered group [36]. However, this study did not find that covering semi-membrane had a significant impact on the degradation of organic matter.

The C/N in the non-covered and covered groups decreased from 15.03 ± 0.54 and 14.97 ± 0.76 to 12.21 ± 0.50 and 12.17 ± 0.53, respectively. The overall C/N showed a downward trend related to the NH_3_ emission during the high-temperature period [37,38]. In Figure 3c, there was no difference in C/N between the upper and bottom layers in the covered versus the non-covered group (*p* for non-covered = 0.207, *p* for covered = 0.151). This indicated that the C/N may not influence the spatial difference in the composting piles.

#### 3.2.3. Germination Index (GI), pH, and Electrical Conductivity (EC)

At the end of composting, the GI was 82.79% ± 8.18% in the non-covered group and 85.82% ± 6.54% in the covered group, which indicated that both composting groups were non-phytotoxic [39,40]. In Figure 3d, a significant difference in GI between the upper and bottom layers was observed in the non-covered group (*p* = 0.093), but not in the covered group (*p* = 0.29). The results show that the membrane may enhance the degradation of VS by increasing the oxygen consumption in the whole pile and, in turn, improve the maturity (evaluated by GI). It can be concluded that the membrane may improve the composting efficiency and spatial homogeneity. 

There was no difference in pH between upper and bottom composting layers during aerobic composting (*p* for non-covered = 0.973, *p* for covered = 0.536). In Figure 3e, in the early and middle stages of composting (Days 4, 12, 16, and 20), the pH in the upper layer in the covered group was higher than that in the bottom layer (on Days 8 and 28, the pH of the bottom layer of the compost was slightly higher due to the influence of composting). The electrical conductivity (EC) showed a steady fluctuation trend ranging from 3.31 ± 0.25 to 3.32 ± 0.34. The EC in the non-covered group was also higher than that in the covered group. In Figure 3f, a significant difference was observed between the upper and bottom layers in the covered and non-covered groups (*p* < 0.001). The EC in the bottom layer in the covered group and non-covered group was higher than that in the upper layer. EC may reflect in the ion level and exchange capacity in the composting process. It was shown that the compost was considered decomposed when EC was lower than 4 ms/cm [41]. The membrane covering had a favorable effect on decomposition in the composting piles. In addition, the changes in EC were associated with NH_3_ emissions and the conversion of NH_4_^+^ and NO_3_^−^ [42].

### 3.3. Oxygen Concentration and Gases Emissions

#### 3.3.1. Oxygen Concentration

**Figure 4:**
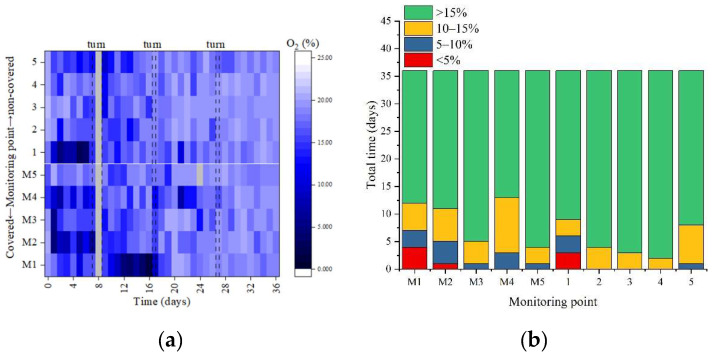
Spatial distribution of oxygen concentration (**a**) and proportion of total time in different oxygen concentration ranges (**b**) at different monitoring points during the aerobic composting. M1–5, the five monitoring points in the covered group.

In Figure 4a, the oxygen concentration in different zones showed significant differences and an increasing trend. 

Compared with the covered group, the non-covered group was “open” to a certain extent, which could provide O_2_ in the upper layer of the composting piles due to air convection [4]. Additionally, the bottom aeration system may make the bottom oxygen concentration higher than the upper layer. Therefore, the spatial oxygen concentration difference in the non-covered group was smaller than that in the covered group. In Figure 4b, during aerobic composting, the average O_2_ concentration in the non-covered and covered groups was 17.46% ± 3.07% and 16.48% ± 3.90, respectively. The anaerobic rate of the stack (<5%) was 1.67% in the non-covered group and 2.77% in the covered groups. This indicated that, with the same aeration volume, the oxygen consumption rate in the covered group was higher compared with the non-covered group. This also demonstrated that the membrane covering was beneficial to the aerobic microbial activity for organic matter degradation. This was also consistent with the abovementioned degradation of VS and the trend and spatial difference performance of GI.

#### 3.3.2. Greenhouse Gases (GHGs), Ammonia (NH_3_) Emissions, and Power Consumption Analysis

**Figure 5:**
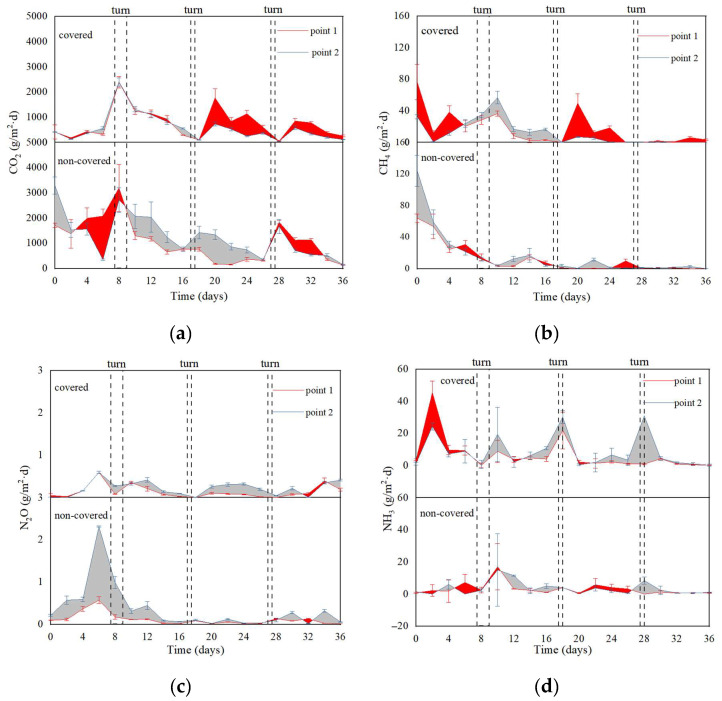
Dynamic changes of CO_2_ (**a**), CH_4_ (**b**), N_2_O (**c**), and NH_3_ (**d**) at different monitoring points during aerobic composting.

In Figure 5a, the emission of CO_2_ was strongly related to the degradation of VS. The larger the degradation rate, the more CO_2_ would be produced. However, the functional membrane has a more obvious barrier effect on CO_2_ flow out [9]. 

The temperature was rapidly increasing with rapid oxygen consumption and CH_4_ was generated [43]. During Days 8–16, the CH_4_ emission rate in the covered group was higher than that in the non-covered group (Figure 5b) and the higher MC in the covered group may promote the forming of anaerobic conditions. Meanwhile, the molecular structure of CH_4_ was smaller and the barrier emission effect of the functional membrane was weaker. In Figure 5c, the N_2_O emission rate in the covered group was lower compared with the non-covered group. N_2_O was also mainly generated in anaerobic conditions by the denitrification process [44]. However, as a macromolecular gas, N_2_O was obviously blocked by functional membranes. In Figure 5d, the NH_3_ emission rate in the covered group was significantly higher than that in the non-covered group. Xiong et al. [11] indicated that the increased emission of ammonia gas by a membrane covering may be related to the formation of a water film under the membrane. It may be explained that ammonium easily dissolves in the water film on the inner layer of the membrane. Moreover, the aeration makes a micro-positive pressure environment form under the membrane and ammonia overflow easily. Furthermore, this might also be explained by the beneficial effect of the membrane covering on nitrogen degradation.

In Table 2, compared with the non-covered group, the cumulative CO_2_ and N_2_O emission in the covered group was reduced by 41.80% and 26.19%, respectively. The cumulative CH_4_ in the covered group was increased by 7.67%. The membrane covering lowered the total GHG emissions by 30.26%, but the NH_3_ emission of the covered group increased by 2~3 times in the non-covered group. The power consumption was 0.26 kWh /m^3^ in the two groups. It was estimated that the power consumption in the composting technology with turning only was 0.33 KWh/m^3^. Thus, aeration with a membrane covering may reduce gaseous emissions and power consumption.

### 3.4. Spatial Distribution of Microbial Community Succession

**Figure 6:**
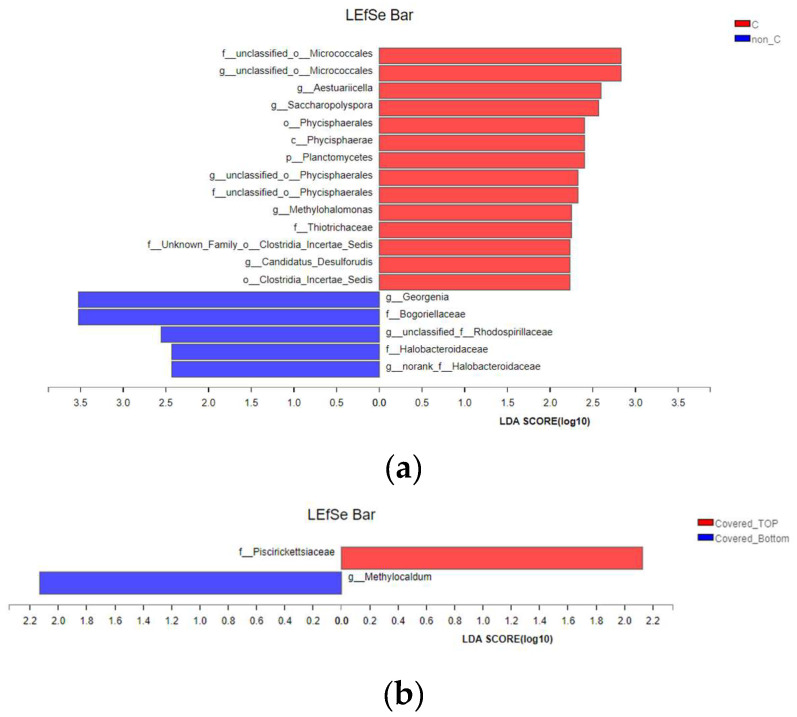

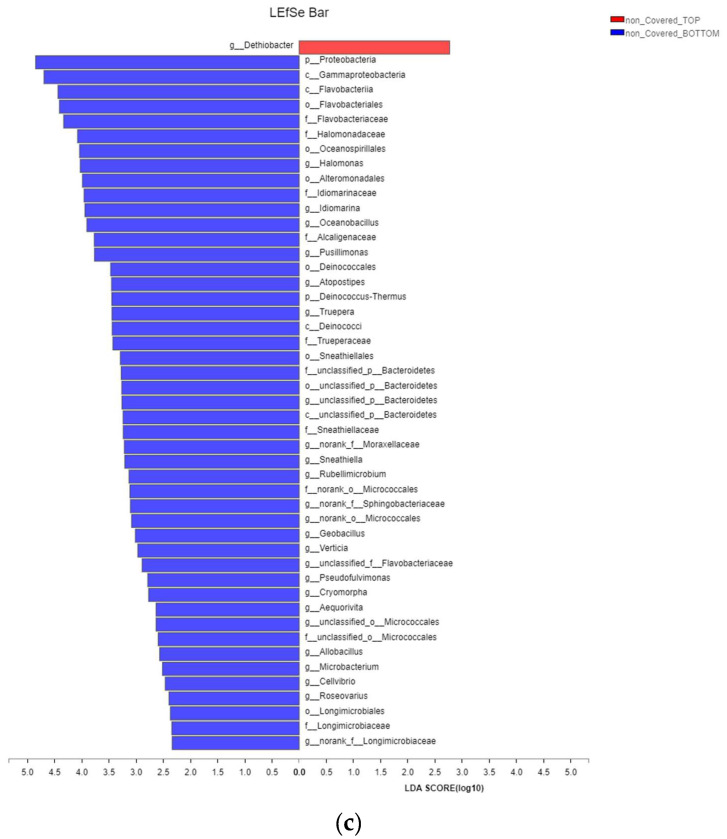
LEfSe multi-level species difference discriminant analysis between the covered and non-covered groups (**a**), between the top and bottom layers in the covered group (**b**) and between the top and bottom layers in the non-covered groups (**c**). The LDA discriminant histogram counts microbial groups that significantly affect multiple groups. The LDA score obtained by LDA analysis (linear regression analysis); the larger the LDA score, the greater the difference in species abundance.

The number of OTUs in the lower layer was higher than that in the upper layer, which indicated that the species richness in the lower layer was higher than that in the upper layer (Figure 6). The upper layer was always close to the surroundings and the lower layer had a higher moisture content; thus, the microbes had a suitable living environment in the lower layer [45]. 

In the samples on Days 0 and 12, there was little difference in the number of OTUs between the covered group and the non-covered group. The samples from Days 24–36 showed that the richness of species in the covered group was higher than that in the non-covered group. The clustering features of the samples were also mainly dominated by the composting period, that is, the clustering features in the samples of a certain day were more significant. At the same time, the samples in the upper layer and lower layer showed certain differences. From the phylum level, *Firmicutes*, *Actinobacteria*, *Bacteroidetes*, *Proteobacteria*, *Chloroflexi*, *Spirochaetae*, *Tenericutes*, and *Deinococcus-Thermus* were found to be predominant in dairy manure composting, accounting for over 90% of community abundance in each sample. Fang et al. [12] also found these phyla in the dairy manure large-scale aerobic composting. Wang et al. [46] indicated that *Firmicutes*, *Bacteroidetes*, *Actinobacteria*, and *Proteobacteria* accounted for a larger proportion of the community and were influenced by physicochemical parameters. *Proteobacteria* and *Actinobacteria* play a crucial role in the cycling of N and C and are the dominant N-fixing bacteria during storage. *Bacteroidetes* are known to take part in the degradation of hemicellulose and cellulose [27]. *Chloroflexi* is associated with the degradation of soluble microbial products and biological nutrient removal process. Su et al. [47] showed that bacteria belonging to the phylum *Chloroflexi* were capable of survival under micro-aerobic and anaerobic conditions. *Chloroflexbacteria* is a facultative anaerobic organism. In the current study, the moisture content in the covered group was higher and the anaerobic environment was more likely to cause a large number of *Chloroflexi*. There were no significant differences at the phylum level between the upper and lower layers or between the covered and non-covered groups. This is mainly because bacterial species in the composting piles were rich.

The aerobic composting process was rich at the genus level, which mainly consisted of *Thermobifida*, *norank_f__Limnochordaceae*, *Halocella*, *Bacillus*, *unclassified_f__Corynebacteriaceae*, *Marinimicrobium*, *Oceanobacillus*, *Truepera*, *Ruminofilibacter*, *unclassified_f__Peptostreptococcaceae*, *Hydrogenispora*, etc. Of those, *Ruminofilibacter* was positively correlated with methane emissions. Considering the richness of species at the genus level, we focused on exploring their differences due to membrane and space. The different microbes of the non-covered group and covered group were analyzed, as shown in Figure 6a, such as *Georgenia*, *unclassified_o_Microcooccales*, *Aestuariicella*, *norank_f_Halobacteroidaceae, Methylohalomonas*, *unclassified_o_Phycisphaerales*, *Candidatus_Desulforudis*, and *unclassified_f_Rhodospirillaceae* at the genu level, *Thiotrichaceae* at the family level, and *Phycisphaerales*, *Clostridia_Incertae_Sedis*, etc. at the order level. Of those, the relative abundance (RA) of *Georgenia*, *norank_f_Halobacteroidaceae* and *unclassified_f_Rhodospirillaceae* in the covered group was lower than that in the control group. Moreover, the RA of *unclassified_o_Microcooccales*, *Saccharopolyspora*, *Phycisphaerales*, *Physiphaerae*, and other flora in the covered group was higher compared with the non-covered group. In Figure 6b, the main differential microbes in the upper and bottom layers in the covered group were *Methlyocaldum* at genus level and *Piscirickettsiaceae* at family level. Additionally, the microbes’ proportion in the bottom layer was high, which was related to the high MC content and soluble salt ions. In the non-covered group, it showed lots of microbes in the lower layer and only *Dethiobacter* was significantly higher in the top layer. Species of this family (mainly genus *Dethiobacter Alkaphilus*) are common in hypersaline soda lake sediments and could drive a number of biogeochemical cycles essential to their survival [48]. In Figure 6c, many microbes in the bottom layer, e.g., *c_Flavobacteriia*, *o_Flavobacteriales*, and *f_unclassifired_p_Bacteroidetes*, etc., which belonged to *Bacteroidota* at the phylum level, had a significantly higher RA than in the top layer. Nakasaki et al. [49] found that *Bacteroidota* survived in anaerobic and high moisture conditions. In addition, the bottom layer had a high moisture content, which was the same result found by Wang et al. [5]. Thus, it indicated that these abundant microbes at the bottom layer were mainly because the higher water content at the bottom was conducive to the survival of the microbes. Besides, in Figure 6b,c, the degree of differences in microbes between the bottom layer and top layer in the non-covered group was greater than those in the covered group. It may be indicated that the membrane could reduce the variability in the spatial structure of the microbes. However, differences in the size and shape of the composting piles in the actual large-scale aerobic composting process might affect the distribution of microbial communities in this space. Further research in this area is needed.

## 4. Conclusions

The spatial distribution characteristics of physicochemical indexes, oxygen concentration, and microbial community distribution in a large-scale aerobic composting system were discussed. A semi-membrane covering enabled the pile to enter the high-temperature period earlier, increased oxygen consumption, and accelerated the maturity of organic matter and compost. The semi-membrane reduced the spatial gradient difference of physical and chemical indexes of the compost pile. In addition, the membrane reduced greenhouse gas emissions and energy consumption. The difference of microbial diversity in the space was reduced by the semi-membrane covering. This study showed that the semi-membrane covering improved the spatial homogeneity and efficiency of fermentation in a large aerobic composting system.

## Figures and Tables

**Table 1:** Composting membrane performance parameter.

Parameter	Units	Type/Value
Functional layer material	-	Polytetrafluoroethylene
Protective layer material	-	Polyester
Unit mass	g·m^−2^	260~450
Breathability at 125 Pa	m^3^·min^−1^·m^−1^	0~0.028
Moisture permeability at 200 Pa	g/m^−2^/h^−1^	0~10^4^
Waterproof	KPa	100
Width tensile strength at 50 cm	kg·mm^−2^	9.3 (radial), 9.7 (latitudinal)
Elongation	%	2.1 (radial), 3.6 (latitudinal)
Water absorption	%	≤8
UV resistance	-	5000

**Table 2:** Comparison of gas emissions and power consumption in two groups.

Group Name	Monitoring Points	CO_2_/(g/m^2^·d)		CH_4_/(g/m^2^·d)		N_2_O/(g/m^2^·d)		NH_3_/(g/m^2^·d)		Power Consumption (KWh /m^3^·Day)
		Maximum	Average	Maximum	Average	Maximum	Average	Maximum	Average	
Covered	Out-membrane 1	2624.16	762.91	98.41	15.38	0.62	0.14	38.32	6.83	0.26
	Out-membrane 2	2553.21	589.28	64.80	11.29	0.62	0.22	52.69	8.93	
Non-covered	1	4137.68	1093.46	69.26	10.99	0.65	0.12	31.34	3.23	0.30
	2	3203.52	1229.82	143.39	13.78	2.33	0.36	37.52	3.64	

## Data Availability

Data used in this study are not available upon appropriate requests to the corresponding author.
